# Validated Simple HPLC-UV Method for Mycophenolic Acid (MPA) Monitoring in Human Plasma. Internal Standardization: Is It Necessary?

**DOI:** 10.3390/molecules26237252

**Published:** 2021-11-29

**Authors:** Paweł K. Kunicki, Aleksandra Wróbel

**Affiliations:** 1 Department of Drug Chemistry, Faculty of Pharmacy, Medical University of Warsaw, Banacha 1, 02-097 Warsaw, Poland; 2 Department of Medical Biology, National Institute of Cardiology, Alpejska 42, 04-628 Warsaw, Poland; a.wrobel@ikard.pl

**Keywords:** HPLC, mycophenolic acid, validation, internal standard, therapeutic drug monitoring

## Abstract

The aim of the work was to prepare a simple but reliable HPLC-UV method for the routine monitoring of mycophenolic acid (MPA). Sample preparation was based on plasma protein precipitation with acetonitrile. The isocratic separation of MPA and internal standard (IS) fenbufen was made on Supelcosil LC-CN column (150 × 4.6 mm, 5 µm) using a mobile phase: CH_3_CN:H_2_O:0.5M KH_2_PO_4_:H_3_PO_4_ (260:700:40:0.4, *v*/*v*). UV detection was set at 305 nm. The calibration covered the MPA concentration range: 0.1–40 µg/mL. The precision was satisfactory with RSD of 0.97–7.06% for intra-assay and of 1.92–5.15% for inter-assay. The inaccuracy was found between −5.72% and +2.96% (+15.40% at LLOQ) and between −8.82% and +5.31% (+19.00% at LLOQ) for intra- and inter-assay, respectively, fulfilling acceptance criteria. After a two-year period of successful application, the presented method has been retrospectively calibrated using the raw data disregarding the IS in the calculations. The validation and stability parameters were similar for both calculation methods. MPA concentrations were recalculated and compared in 1187 consecutive routine therapeutic drug monitoring (TDM) trough plasma samples from mycophenolate-treated patients. A high agreement (r^2^ = 0.9931, *p* < 0.0001) of the results was found. A Bland–Altman test revealed a mean bias of −0.011 μg/mL (95% CI: −0.017; −0.005) comprising −0.14% (95% Cl: −0.39; +0.11), whereas the Passing–Bablok regression was y = 0.986x + 0.014. The presented method can be recommended as an attractive analytical tool for medical (hospital) laboratories equipped with solely basic HPLC apparatus. The procedure can be further simplified by disapplying an internal standard while maintaining appropriate precision and accuracy of measurements.

## 1. Introduction

Mycophenolic acid (MPA) is an immunosuppressive agent presenting high inter- and intra-subject pharmacokinetic variability. It is available either as inactive pro-drug mycophenolate mofetil (MMF), or as enteric-coated mycophenolate sodium (EC-MPS) [1,2,3]. MPA is an important constituent of several immunosuppressive maintenance protocols being co-administered mainly with calcineurin or m-TOR inhibitors. Published results of concentration-controlled trials support a theory that therapeutic drug monitoring (TDM) may be beneficial for guiding MMF dosing in clinical practice [3,4,5]. Steady-state trough C_min_ determination is still widely used, although it has proven to be a limited predictor of drug exposure [3]. The AUC parameter (mainly: AUC_0–12h_), better correlated with the clinical effect, has all the disadvantages of multiple drug determinations, hence the leading centers have been using a limited sampling strategy (LSS) and calculating AUC_0–12h_ values based on the algorithms obtained for the therapeutic scheme used in the center [3,4,5]. The use of AUC-based monitoring requires the determination of MPA concentrations several times higher than with C0 monitoring. Immunosuppressive poly-therapy may lead to pharmacokinetic interactions affecting MPA concentration [3]. MPA is extensively biotransformed into the inactive glucuronide (MPAG) observed in concentrations many times exceeding MPA concentration in plasma, and into the active acyl-glucuronide (AcMPAG) which participates in small extent in pharmacodynamic effect of the drug [2,3]. It is also discussed that monitoring free fraction of MPA may be helpful for some groups of patients, e.g., with hypoalbuminemia or kidney impairment [2,3]. Despite of the incessant interest in the analysis of MPA metabolites and/or free MPA, it still is the total MPA concentration which is predominantly determined for routine TDM purposes [2,4]. Therefore, reliable analytical methods are needed to measure total MPA concentration in validated range adequate for both trough samples (including MPA monitoring during interactions) and pharmacokinetic profiling. There is also a need for the determination of MPA in pharmacokinetic studies.

Currently, the techniques for determining MPA in plasma can be divided into four types: two groups of chromatographic methods (considered as reference), among which we can distinguish (1) chromatography with mass detection (LC-MS/MS) and (2) classic HPLC or UHPLC coupled with UV or, less frequently, fluorescence detection, further: (3) immunoassays (i.e., EMIT, PETINIA, CEDIA) and also (4) IMPDH Inhibition Assay from Roche [2,3]. Techniques 3 and 4 have the advantage of being available in an automated form, making them preferred in small and/or local clinical laboratories serving transplantation TDM. Chromatographic techniques are currently preferred at larger TDM centers employing highly educated and trained analysts, often located at academic hospitals. It is generally well recognized that a properly validated chromatographic method is superior to immunoassay because of its specificity [2,3,6,7]. Contrary to calcineurin or mTOR inhibitors, because of relatively higher (in µg per mL) plasma concentrations, MPA is not doomed only to advanced LC-MS and inexpensive UV detection is perfectly sufficient [3,6,8,9]. Since the 1990s, a number of chromatographic methods for the determination of MPA in human plasma have been published. These methods have been compared in several reviews [7,8,10,11]. MS or tandem MS/MS detection was applied in a list of publications including multi-drug methods [12,13,14,15,16,17,18,19,20,21], and there are also papers with fluorescent detection [22,23]; however, UV spectroscopy is a most frequently used detection [6,9,10,24,25,26,27,28,29,30,31,32,33,34,35,36,37,38,39,40,41,42,43,44,45,46,47,48,49,50]. Most methods used C8 or C18 packed columns, and detection was commonly carried out at a wavelength of ca. 210 or 254 nm. Internal standardization was implicitly accepted procedure for analytical HPLC-UV methods. Early methods are characterized by inconvenient preparation procedures [26,33,34,35,38,46,48,49], demanding chromatography including gradient elution [24,35,36,37,38], relatively long analysis runtime [10,25,26,27,31,34,37,39,40,42,43,44,45,47,49], large sample volumes [33,39,40,44,45,46,49]. Other methods were poorly calibrated or validated [10,27,28,29,30], or the obtained validation parameters are insufficient as expected for TDM [32,36,38,41,47]. None of the previous HPLC-UV methods guaranteed a sufficiently broad analytical range for TDM with a runtime of less than 10 min and an uncomplicated sample preparation procedure.

The original aim of the work was to prepare an analytical method for the routine monitoring of MPA, which would be an alternative, on the one hand, to LC-MS/MS, which is expensive to obtain and requires appropriate skills, and, on the other hand, to not very specific, and therefore not very reliable, immunochemical techniques. Our goal was therefore a simple but reliable method, easily achievable for a hospital TDM laboratory. Uncomplicated protein precipitation was selected for sample preparation, with subsequent chromatography on cyanopropyl derivatized silica phase (CN) which has been positively verified for other drugs but hardly ever used for MPA. For detection, a higher analytical wavelength (ca. 305 nm) was chosen which is expected to significantly reduce chromatographic interference. After a two-year period of successful application, confirmed by good results from the Mycophenolate International Proficiency Testing Scheme (Analytical Services International, London, UK), the presented method was retrospectively investigated to assess the real need for calibration based on the use of an internal standard, which became an additional aim of the work.

## 2. Results

### 2.1. Method Development

#### 2.1.1. Extraction

The use of acetonitrile for deproteinization of plasma samples is a simple and rapid stage of preparing the samples for HPLC. That is especially attractive for drugs presenting relatively high (in µg per mL) plasma concentrations. Consequently, we could use such a procedure for MPA determination. The absolute recovery was analyzed by comparing the peak areas for extracted calibration standards with those obtained from direct injection of equivalent quantities of standards taking into account the volume ratio. The analytical procedure based on plasma protein precipitation resulted in satisfactory recoveries yielding: 86.04 ± 3.54% (*n* = 38) being stable in the whole concentration range tested (80.75–87.34%) for MPA and 83.01 ± 2.76% (*n* = 38) for IS.

#### 2.1.2. Chromatographic Separation

**Figure 1:**
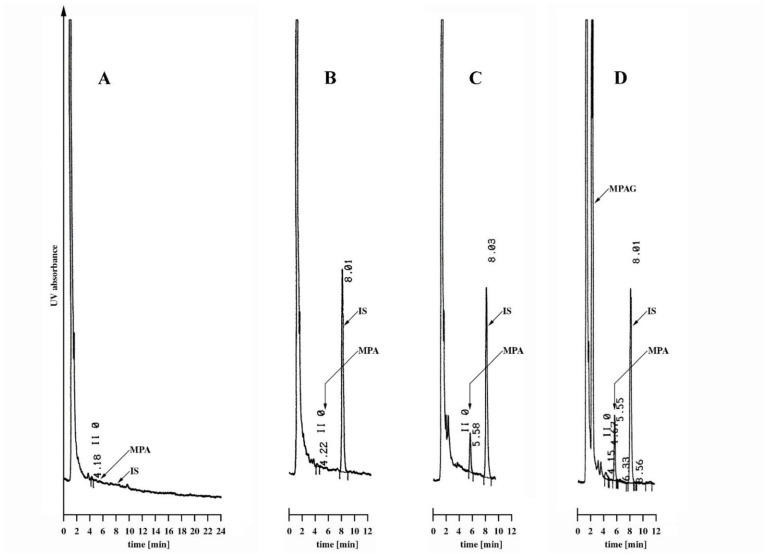
Chromatograms of plasma samples analyzed as described in Materials and Methods (signal attenuation 16): (**A**) drug-free plasma analyzed without IS, (**B**) drug-free plasma, (**C**) drug-free plasma spiked with MPA to obtain the concentration of 1 µg/mL, (**D**) plasma sample taken from the patient treated with MMF containing 1.53 µg/mL of MPA. Peaks: MPAG: ~2.2 min, MPA: ~5.6 min, IS: ~8.0 min.

The optimal acetonitrile content, as well as the phosphate concentration in the mobile phase, were selected experimentally. Under the analytical conditions presented in Section 4.3, the LC-CN column provided complete separation of MPA from both MPAG and fenbufen (IS) and a low, clean background of the biological sample (Figure 1). Described mobile phase composition guaranteed satisfactory repeatability of the retention times (RTs), which were 2.2 min, 5.6 min and 8.0 min for MPAG, MPA and IS, respectively. With no intention of quantifying MPAG, we did not strive to separate its peak from the components of the biological matrix. This can easily be achieved by modifying the composition of the mobile phase.

### 2.2. Method Validation

#### 2.2.1. Specificity

As previously reported, MPA and IS were well separated from each other, showing peaks of good symmetry shape without a significant tailing effect. Twenty plasma samples randomly drawn (using K_2_EDTA blood collection tubes) from cardiac patients not treated with MMF were analyzed and evaluated for interference. No significant interference neither with plasma matrix constituents nor with co-administered drugs was noted under finally established chromatographic conditions. An exemplary chromatogram is shown in Figure 1A.

#### 2.2.2. Calibration and Linearity

The linearity of the detection system response was assessed injecting from 5 to 2000 ng of MPA onto the column using specially prepared MPA solutions covering the concentrations of 0.1–40 µg/mL in plasma, as described in Section 4.4. The detector response for MPA was perfectly described by the straight-line equation (y = 4172x − 5269) in the entire studied concentration range with the high value coefficient of determination r^2^ = 0.9999. 

Having confirmed the response from the UV detector, the method was calibrated and found linear up to 40 µg/mL. The calibration curves were obtained by analyzing plasma samples for each of eight concentrations tested, i.e., 0.1–0.5–1–2–5–10–20–40 µg/mL on four different analytical runs, the first in quintuplicate, and the next in duplicates. Due to observed heteroscedasticity, the curves were calculated by a weighted linear regression analysis with w = 1/x implemented for improving adjustment at low concentrations. The four calibration curves obtained were linear and described by following Equations (1)–(4):

MPA = 5.9390 × F + 0.01620, r^2^ = 0.9992, (1)

MPA = 5.9979 × F + 0.02456, r^2^ = 0.9996, (2)

MPA = 6.1194 × F + 0.00386, r^2^ = 0.9997, (3)

MPA = 5.8343 × F + 0.01336, r^2^ = 0.9998, (4)

where: MPA stands for mycophenolic acid concentration in µg/mL, and F is a factor obtained from peak areas: MPA/IS.

The calculation of combined data from all calibration curves led to the final algorithm: MPA concentration [μg/mL] = 5.9719 × F + 0.01445.

#### 2.2.3. Precision and Accuracy

To assess the precision and accuracy of the method, MPA concentration measurements from the calibration curves were used (Section 2.2.2). The intra-assay evaluation was based on the first calibration (*n* = 5), while the data from all calibrations was used in the inter-assay evaluation (*n* = 4). Furthermore, three levels of control samples (L, M, H, *n* = 3, Section 4.4) were also used for inter-assay. Detailed information is given in Table 1. In addition to the results obtained (according to the procedure) using IS, this table includes, for comparison, also the results obtained from calculations without the use of the internal standard. For clarity, for each concentration level, the results obtained with the use of IS are shown in the upper row while those calculated without the use of IS are shown in the lower row just below. The precision calculated using IS was satisfactory in the whole range tested with relative standard deviation (RSD) of 0.97–7.06% for intra-assay and of 1.9–5.15% for inter-assay. The accuracy of the method was calculated using the data from precision testing. The intra-assay inaccuracy and the inter-assay inaccuracy calculated using IS was found between −5.72% and +2.96% (+15.40% at LLOQ) and −8.82% and +5.31% (+19.00% at LLOQ). The results fulfilled EMA and FDA requirements [51,52].

#### 2.2.4. Limit of Quantification, Range, and Carry-Over

Lower limit of quantification (LLOQ) parameter was the lowest calibration standard with acceptable accuracy and precision (Table 1). LLOQ was set at 0.1 µg/mL. The calibration covered MPA concentrations up to 40 µg/mL. The carry-over effect was detected by injecting extracted blank samples (drug-free) after the upper limit of quantification (ULOQ) sample. No carry-over effect was observed.

### 2.3. Stability

The stability of analytical method should be checked to ensure that the storage conditions as well as every step taken during sample preparation and sample analysis do not affect the concentration of the analyte [51]. The method stability was confirmed in a series of experiments including freeze-thaw and short- and long-term stability testing. The results are presented in Table 2, Table 3 and Table 4. Similar to the content of Table 1, these tables include, for comparison, also the results obtained from calculations without the use of the internal standard presented in the bottom row of each cell.

#### 2.3.1. Long-Term Stability

In the long-term stability test, measurements were taken immediately after preparation of spiked sample (for recording initial MPA values), which were then aliquoted and stored frozen at −24 °C; for a period of six weeks. The set of three samples for both test levels (low and high) was thawed every seven days and upon reaching ambient temperature the MPA concentration was determined. The stability of the analytes in plasma after long-term storage (six weeks) at −24 °C was found satisfactory. The detailed results are presented in Table 2.

#### 2.3.2. Freeze-Thaw Stability

Freeze-thaw stability was done both with low (~1.2 µg/mL) and high (~25 µg/mL) MPA plasma samples prepared from drug-free plasma. The samples were determined as described, placed in the freezer (at −24 °C) and subsequently analyzed after 72, 144, and 216 h. After thawing, the samples were frozen again in the same conditions. The data given in Table 3 proved MPA stability during the test.

#### 2.3.3. Short-Term Stability

Plasma samples with MPA added to reach the concentrations ~1.2 and ~25 µg/mL were analyzed with the standard analytical procedure (*n* = 3) for testing short-term MPA stability in plasma. First, the standard analytical course for six simultaneously prepared samples was conducted in accordance with sample preparation procedure without interrupting step. Second, supernatant stability was tested in Eppendorf test tube left for 4 h after centrifugation also in 6 simultaneously prepared samples. After that, the stability of dried extract in a glass tube left for 4 h after evaporation at room temperature was tested also in six samples prepared similarly. After storage at that stage, the procedure was resumed, the samples reconstituted and injected onto the column. Finally, the standard analytical course was interrupted just before injection for another six samples, which were injected onto HPLC after 4 h of resting reconstituted at ambient temperature. The results are included in Table 4. No significant changes were noted.

### 2.4. Comparison of the Measures for Internal and External Standardization

As provided in Section 4.8. Internal and External Standardization, a retrospective evaluation was performed in the case of a decision to calibrate the method based on the so-called external standardization, without using of IS. Data from calibration curves (Section 2.2.2. Calibration and Linearity) obtained for method validation were used. The calculation of combined data from all calibration curves led to the algorithm: MPA concentration [μg/mL] = MPA peak area/35,302 + 0.01617. This formula was used to calculate MPA concentration in describing precision, accuracy and stability, and in patient and QC samples. Validation parameters without the use of IS were obtained exactly for all the same measurements as in the original validation (results presented in Section 2.2 and Section 2.3).

#### 2.4.1. Validation Parameters

The precision calculated using no IS was also satisfactory in the whole range tested with relative standard deviation (RSD) of 0.90–8.42% for intra-assay and of 0.48–6.09% for inter-assay. The intra-assay inaccuracy and the inter-assay inaccuracy calculated using no IS was found between –3.59% and +2.21% (+14.30% at LLOQ) and –7.81% and +2.27% (+18.72% at LLOQ). Detailed information is given in Table 1. The numerical results corresponding to the measurement without the use of IS are shown in the additional bottom row while the results obtained with the IS are shown in the upper row above. Overall, the accuracy and precision results were similar for both calculation methods.

#### 2.4.2. Stability Results

The stability results obtained from calculations without the use of the internal standard are included in Table 2, Table 3 and Table 4. For easily comparison the numbers are presented (as in Table 1) in the bottom row of each cell. The analysis of the results presented in the tables showed that MPA concentrations calculated without the use of IS confirm the required stability as well. Moreover, the deviation of the measured concentration from the baseline value seen in stability tests was lower when it came from calculations without using of the internal standard. Possible causes are discussed later in this paper.

#### 2.4.3. Patient’s Samples

**Figure 2:**
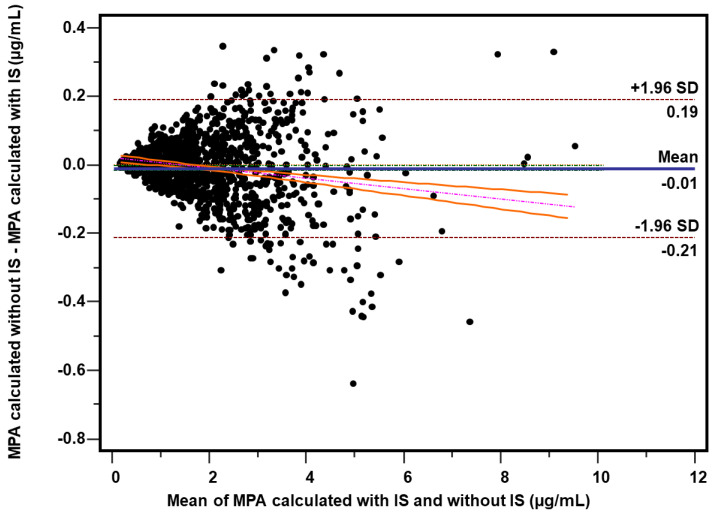
Comparison of calculation methods made on a total of 1187 samples from heart transplant patients. Bland–Altman analysis—plot of difference against mean for MPA concentrations (µg/mL) obtained without using IS and with using IS presented as absolute bias. Horizontal lines represent bias (solid: mean, dashed: ±1.96 SD), whereas the regression line of differences (with its 95% CI) is indicated by dotted line.

**Figure 3:**
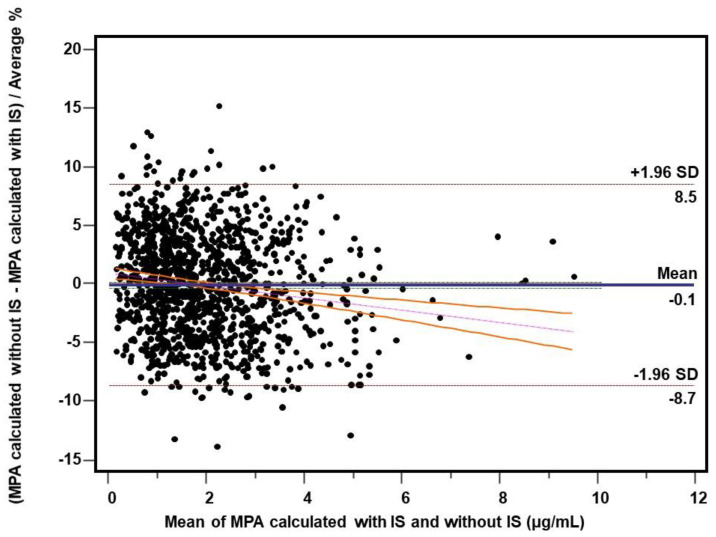
Comparison of calculation methods made on a total of 1187 samples from heart transplant patients. Bland–Altman analysis—plot of difference against mean for MPA concentrations (µg/mL) obtained without using IS and with using IS presented as percent value. Horizontal lines represent bias (solid: mean, dashed: ±1.96 SD), whereas the regression line of differences (with its 95% CI) is indicated by dotted line.

**Figure 4:**
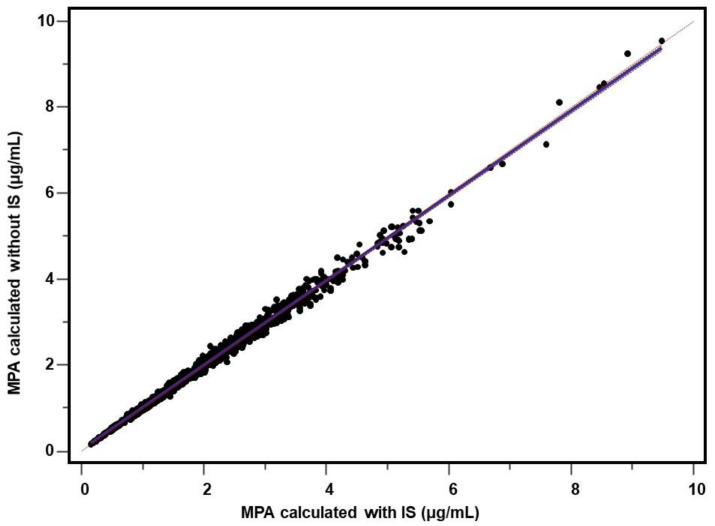
Comparison of calculation methods made on a total of 1187 samples from heart transplant patients. Passing–Bablok regression: y = 0.986x + 0.014 represents MPA concentrations (µg/mL) obtained without using IS against those obtained with using IS.

MPA concentrations were recalculated using the formula provided in Section 2.4 in 1202 consecutive routine trough (C0) plasma samples from mycophenolate-administered heart transplant patients. The two calculation methods were statistically compared. Due to the fact that 15 samples (1.2%) presented significant interference with the peak of the internal standard fenbufen, the final comparison was made for 1187 measurements. A high agreement of the C0 results was found, confirmed by both the comparison of the means (1.99 ± 1.24 vs. 1.98 ± 1.22 µg/mL, *p* = 0.0154, Wilcoxon test) and the value of the coefficient of determination (r^2^ = 0.9931, *p* < 0.0001). Data are included in Table 5. Bland–Altman test revealed mean bias of −0.011 μg/mL (95% CI: −0.017; −0.005) comprising −0.14% (95% Cl: −0.39; +0.11) (Figure 2 and Figure 3) whereas Passing–Bablok regression was y = 0.986x + 0.014 (95% Cl for slope: 0.981; 0.992 and for intercept: +0.007; +0.021) (Figure 4).

#### 2.4.4. Spiked Samples

A total of 90 drug-free plasma spiked MPA at low (~1.2 µg/mL, *n* = 45) and high (~25 µg/mL, *n* = 45) concentrations taken from stability studies were recalculated. The results are presented in Table 6. The mean values for both calculation methods (with IS vs. without IS, respectively) were very similar for both low (1.23 ± 0.07 vs. 1.21 ± 0.05 µg/mL) and high (25.24 ± 1.05 vs. 25.14 ± 0.69 µg/mL) MPA concentrations.

## 3. Discussion

The presented method for determining MPA concentration in plasma is characterized by satisfactory validation parameters, which make it suitable for use in routine drug monitoring. The obtained parameters make the method also applicable in MPA pharmacokinetic studies. Several validation parameters are particularly relevant to the methods intended for therapeutic drug monitoring. Specificity is fundamental to ensure that only the desired substance is determined. In the case of MPA, only chromatographic techniques (which include our method) and the IMPDH Inhibition Assay from Roche allow the appropriate specificity of the measurement [3]. Another important parameter characterizing the method is its range. In our case, the range from 0.1 to 40.0 μg/mL allows the method to be used both for monitoring C0 (concentrations usually <10 μg/mL) and for full or shortened pharmacokinetic profiles (concentration values up to ~30–40 μg/mL, data not shown), and also in case of pharmacokinetic interactions and pre-laboratory errors when C0 is monitored. In this regard, neither the immunochemical methods nor the IMPDH Inhibition Assay provide a comparably broad range that can only be offered by chromatographic techniques [3,53]. The range of the analytical method is defined by the precision and accuracy of the measurements made. The EMA and FDA guidelines define imprecision and inaccuracy to be ≤15% and ≤20% at LLOQ [51,52]. According to the current IATDMCT recommendations included in the latest consensus on MPA monitoring we aim for LLOQ ≤0.2 µg/mL, inter-assay imprecision ≤10%, preferably ≤5%, and analytical bias (inaccuracy) ≤10%, preferably ≤5% [3]. The method developed by us meets the above EMA and FDA criteria in the entire concentration range: 0.1–40.0 µg/mL, at the same time for the MPA concentration of 0.3 µg/mL (QC-L), the intra-run imprecision did not exceed 5.15%, and the intra-run inaccuracy did not exceed 8.82% (Table 1), which satisfactorily matches the method to the consensus requirements [3,51,52,53]. Complementing the validation was stability confirmation performed in the EMA recommended long-term stability, freeze-thaw stability and short-term stability tests detailed in Table 2, Table 3 and Table 4. It can be considered that the validation parameters (keeping in mind the specificity of the method) are at the level obtained when using the LC-MS/MS technique.

In accordance with the assumed aim of the work, apart from ensuring appropriate validation parameters, the developed chromatographic method was to be an attractive analytical tool. We used a simple, conventional HPLC technique with UV detection. The system consists of modules: the pump works in an isocratic mode, the analyses are performed at room temperature with separation on the classic LC-CN column, known for many years, using a mobile phase with a relatively simple composition. Detection is performed at a single wavelength. We did not use an autosampler in the system, but a simple manual injector. This can be considered a drawback or an advantage—on the one hand, robustness may be improved by the use of autosampler, which is now a standard component of the HPLC system; on the other hand, with a small number of analyzes in a series of less than 10—the use of manual sample injection is quite sufficient. Simple yet efficient enough is the sample preparation procedure based on deproteinizing the plasma sample—it does not require any advanced apparatus (vortex, laboratory centrifuge, and solvent evaporation unit are easily available even in a basically equipped laboratory). The purpose of supernatant evaporation was to increase the purity of the injected solution and, consequently, to extend the life of the column.

HPLC-UV methods assume detection at a single wavelength consistent with the characteristic absorption maxima of the MPA spectrum, i.e., 215 nm, 251 nm, 304 nm [3,10,34,35,37,41,42]. The spectrum can be easily found, for example, in the publication of Daurel-Receveur et al. [35]. We compared the MPA absorbance at points corresponding to successive wavelength maxima using the UV detector applied in our research. The signal for MPA at 305 nm was 1.98 times lower than that measured at 251 nm and 9.14 times lower than that measured at 215 nm. These results are fully consistent with those obtained by Shipkova et al. [37], Wiwattanawongsa et al. [34], Daurel-Receveur et al. [35], and Chen et al. [42]. Most of the researchers chose one of the first two maxima for the analytical wavelength, where the MPA signal intensity is higher. However, the polytherapy common to immunosuppressive therapy can cause chromatographic interference when using these wavelengths. Unlike most authors, we chose to measure at λ = 305 nm. Interferences are much less under these conditions, and the MPA signal is intense enough to ensure adequate analyte quantification [35,42]. It can be assessed that for the price of a lower MPA signal, we obtained a high chromatographic purity of the sample, which confirms the lack of interference at MPA retention time; only a negligible (1.2%, 15 of 1202) percentage of samples from just a few patients presented an interference with the peak of internal standard.

In the chromatographic separation, we used a column with a cyanopropyl derivatized silica phase (LC-CN) that has been known for many years. Under the analytical conditions presented, the LC-CN column provided a good separation of MPA from both MPAG and fenbufen (IS) and a low, clean background of the biological sample (Figure 1). Retention times were acceptably short at 5.6 min (MPA) and 8.0 min (IS), allowing one run to be finished in 10 min; further reduction of the runtime is also possible. In our practice, the Supelcosil LC-CN column protected by the Supelguard precolumn provided stable chromatographic parameters for 1500–2000 injections of extracted samples, which corresponded to approximately two years of operation. Previous publications have mostly used columns with classic C18 or C8 packing. The use of LC-CN column for MPA determination was described previously by Westley et al. [31]. Compared to our method, the analysis time is longer (up to 14 min), the calibration range is narrower (0.25–20.0 µg/mL), and the internal standard (carboxy butoxy ether mycophenolic acid) is more difficult to achieve. The second method in which an LC-CN column was used (as well as detection at 304 nm) was the work of Sugioka et al. [49]. The disadvantages of the procedure are the complicated, multi-stage extraction with the use of large volumes of reagents, carried out with as much as 1 mL of a plasma sample, a long 30 min run-time and a low extraction efficiency of approx. 50%.

Fenbufen used by us has not been previously described as a potential IS for MPA determination, and it is an easily available, has a suitable UV spectrum, good stability and it was withdrawn from the markets of developed countries in 2010, which is an advantage from an analytical point of view [54]. The validation parameters presented in Table 1 confirm the reliability of the method calibrated using fenbufen as an internal standard. During approximately two years of practical use of the method for monitoring MPA concentration, we observed only sporadic cases of interference at the retention time of IS. The interference with fenbufen has become a pretext to evaluate the methodology when resigning from the use of IS and the calculation is based on the calibration: MPA signal (peak area)—MPA concentration. Using the experimental (raw) data from repeated calibrations of the method, a formula was calculated based on the peak area measurement. Using this formula, all relevant validation parameters were recalculated, i.e., precision and accuracy (Table 1) and stability (Table 2, Table 3 and Table 4). For the sake of clarity of the comparison, the numerical results corresponding to the measurement without the use of IS are shown in tables in the additional bottom row while the results obtained with the IS are shown in the upper row above. As can be seen, the precision and accuracy determining the range of the method are comparable regardless of whether or not IS is used. Using this approach, MPA concentrations were recalculated in 1202 consecutive routinely determined plasma samples from patients treated with mycophenolates. Similarly, 90 samples of drug-free plasma spiked MPA at low (~1.2 µg/mL) and high (~25 µg/mL) concentrations were also recalculated. The comparison of the C0 results (*n* = 1187) showed a very high agreement (r^2^ = 0.993, *p* < 0.0001) between both calculation methods. The Bland–Altman test (Figure 2 and Figure 3) revealed only a minimal bias of −0.011 µg/mL and −0.14%. Due to the relatively large number of measurements, these minimal differences turned out to be statistically significant both in the Bland–Altman test, in the Passing–Bablok regression (Figure 4) or in the comparison of the means: 1.99 vs. 1.98 µg/mL (*p* = 0.0154). However, with regard to the therapeutic MPA concentration values, it should be firmly stated that the differences found are of no practical significance in the clinical evaluation of the outcome. Similar conclusions can be taken from the analysis of spiked samples—the mean values for both calculation methods were very similar for both low (1.23 vs. 1.21 µg/mL) and high (25.24 vs. 25.14 µg/mL) MPA concentrations. It is worth noting, however, that unexpectedly the measurements performed without IS are characterized by a lower variability than the measurements with the use of IS: 4.55% vs. 5.91% and 2.73% vs. 4.18% for low and high MPA concentrations, respectively. Paradoxically, the greater variability for the determinations when IS was used may be partly due to the additional step of manually dispensing the IS working solution with a Hamilton syringe. Despite the greatest care, the step of dispensing an internal standard into a sample will always be a potential source of error related to both the volume dispensed and the slight variation in the concentration of the IS working solution. This phenomenon can be seen in the results of the stability study. Especially in the long-term and freeze-thaw tests (Table 2 and Table 3), the deviation of the measured concentration from the baseline value is higher when it comes from the IS based calculations. The fluctuation of the measured MPA concentration can be partially explained by a potential slight (accepted up to 3%) change in the concentration of the IS working solution during storage. An alternative is to calibrate the method for each analyzed series of samples, which is recommended for laboratories performing a large (several dozen or more) daily number of MPA samples. For a laboratory with less than a dozen determinations a day, a practical solution is to control the concentration of the IS working solution and, of course, regular analysis of QC samples. Participation in the proficiency testing scheme is fully recommended [3,55]. Calibration of the method should be performed as needed, in accordance with the laboratory’s procedures and quality assurance policy. 

Our results proved that sometimes for the simple HPLC-UV method it is not necessary to use IS, and even that it is better not to use it. Such a finding undermines the common belief that the method with IS is more valuable and reliable and that internal standardizing is the procedure of choice. Of course, the use of an appropriately selected IS allows compensation for loss in sample volume during the analysis, and generally allows the sample to be controlled during the determination. Looking at these benefits, the analyst often does not see the fact of introducing an additional substance to the chromatographic analysis, which necessitates an extra step in the analytical procedure with the requirement of accurately injecting IS into the biological matrix. It also carries the risk of interference between IS and compounds contained in the sample (other drugs, metabolites, endogenous substances). Therefore, in order not to require IS in the routine method for TDM—the analyzed drug (analyte) must be isolated from the biological matrix to a high percentage, and the precision of determinations must be high (imprecision low). In our case, the MPA extraction efficiency was 86.04%, being stable in the entire range of tested concentrations (80.75–87.34%) and this value turned out to be sufficiently high. Internal standard was also not used in several other published methods for the determination of MPA. In the work of Pastore et al. [30] a gradient elution was applied and the validation parameters were described very laconically. In the paper published by Hosotsubo et al. [41] despite simple preparation of the sample with acceptable (<8 min) runtime, the range of the method (0.5–40 µg/mL) was too narrow for actual TDM requirements [3,55]. Hosotsubo et al. argued that since the sample preparation was only protein precipitation with acetonitrile, therefore, calibration with IS was unnecessary [41]. In our opinion, the mere fact of deproteinization is not sufficient to ignore IS and must be confirmed by the previously discussed efficiency of the analyte isolation from the matrix leading to satisfactory validation followed by passed QC and successful participation in proficiency testing. If we ensure that these conditions are met, then, as in our case, a method without an internal standard can be used in routine clinical practice.

## 4. Materials and Methods

### 4.1. Chemicals

Mycophenolic acid (≥98% purity) was obtained from Sigma Aldrich Co. (St. Louis, MO, USA). Fenbufen (IS) was purchased from ICN Biomedicals Inc. (Aurora, OH, USA). HPLC-grade acetonitrile, methanol and water were obtained from Avantor Performance Materials (Gliwice, Poland), orthophosphoric acid 85% was from Chempur (Piekary Śląskie, Poland), and KH_2_PO_4_ was from Merck (Darmstadt, Germany).

### 4.2. Instrumentation

The HPLC isocratic system (Spectra-Physics, San Jose, CA, USA) consisted of a pump (Model P 100), a UV detector (UV 150), an injector with 50 µL loop (Model 7125i, Rheodyne, Cotati, CA, USA), and an integrator (Chrom Jet 4400). Universal laboratory centrifuge (5417 C, Eppendorf, Hamburg, Germany), a vortex-shaker (Reax top, Heidolph Instruments, Schwabach, Germany), and a water bath (LW 502, AJL Electronic, Cracow, Poland) were used for sample preparation.

### 4.3. Chromatographic Conditions

The separation of compounds was made on Supelcosil LC-CN column (150 × 4.6 mm, 5 µm) protected with Supelguard LC-CN precolumn (20 × 4.6 mm, 5µm), both from Supelco Analytical, Bellefonte, PA, USA. The mobile phase was a mixture of CH_3_CN:H_2_O:0.5M KH_2_PO_4_:H_3_PO_4_ (260:700:40:0.4, *v*/*v*). The isocratic flow rate was fixed at 1.5 mL/min. All analyses were performed at ambient temperature. UV detection was set at a wavelength of 305 nm.

### 4.4. Stock and Working Solutions, Calibration, and Quality Controls

Stock solutions of mycophenolic acid (2 mg/mL) and fenbufen (1 mg/mL) were prepared by dissolving appropriate amounts of chemically pure substances in methanol. The MPA working solutions for calibration and controls were prepared from the stock solution by adequately diluting in methanol. Working solutions were added to drug-free plasma to obtain the MPA concentration levels of 0.1–0.5–1–2–5–10–20–40 µg/mL (calibration) and of 0.3–4–25 µg/mL (quality controls). Internal standard (fenbufen) working solution of 15 µg/mL was prepared from the stock solution by adequate dilution in methanol. For the purpose of evaluating the response from the detection system—MPA methanolic solutions with (10× lower) concentrations of 0.1–0.5–1–2–5–10–20–40 µg/mL were made from stock solution allowing the loading from 5 to 2000 ng of MPA onto the column, which corresponds to the target calibration range of 0.1–40 µg/mL in plasma. Drug-free plasma was obtained from healthy volunteers using K_2_EDTA blood collection tubes.

### 4.5. Sample Preparation

A total of 200 µL of plasma was transferred to a 1.5 mL standard Eppendorf tube, first mixed with 20 µL of methanol, and vortexed for 10 s, second mixed with 20 µL of IS working solution, and also vortexed for 10 s. Next, 400 µL of acetonitrile was added to precipitate proteins and the sample was again vortexed for 30 s. After centrifugation (15,000 g, 10 min at ambient temperature), a 200 µL volume of supernatant was transferred to a 10 mL Pyrex conical glass tube and evaporated to dryness in a water bath at 37 °C under a stream of argon. Then the dried extract was reconstituted in 100 µL of mobile phase and an 80 µL aliquot was injected onto the column. The presented procedure was experimentally recognized as optimal and robust.

### 4.6. Method Validation

The method was validated based on the guidelines of the European Medicines Agency (EMA) from 2011 [51]. Particular attention has been paid to specificity, calibration, linearity, range with LLOQ, accuracy, and precision as well as stability. The performed validation also meets the criteria included in the Bioanalytical Method Validation. Guidance for Industry (FDA 2018) [52]. Detailed aspects of the determination of particular parameters are described in the presentation of the results in Section 2.2. Method Validation.

### 4.7. Stability

The stability of the method was assessed in accordance with the recommendations contained in the EMA [51] guidelines with modifications reflecting the requirements for the application of the method for the purposes of TDM. The correction concerned, inter alia, concentration levels that were matched with low and high concentrations of MPA measured in real patient plasma samples. Stability tests were performed using drug-free plasma to which MPA was added at low (L, ~1.2 µg/mL) and high (H, ~25 µg/mL) concentrations. Stability tests most adequate to the actual conditions of the application of the developed method were carried out. Therefore, the following were checked: long-term stability during 6-week storage of plasma samples, freeze-thaw stability, and stability during possible breaks in the analytical procedure (short-term stability). Details of the stability tests methodology are described in the presentation of the results in Section 2.3. Stability.

### 4.8. Internal and External Standardization

Calibration of the developed methodology was based on the application of an internal standard. Fenbufen was used, a compound which, due to its chromatographic properties, seemed to be the optimal candidate as IS. After a 2-year period of routine use of the method, a retrospective evaluation of the calibration, validation, stability and diagnostic application was performed in case of resignation from the use of IS and the calibration based on the so-called external standardization. MPA concentrations were recalculated in 1202 consecutive routinely determined plasma (K_2_EDTA) samples from heart transplant patients treated with mycophenolates as well as in samples of drug-free plasma spiked MPA at low (~1.2 µg/mL, *n* = 45) and high (~25 µg/mL, *n* = 45) concentrations taken from stability studies. The two calculation methods were statistically compared using Passing–Bablok regression, linear regression model and Bland–Altman procedure adequate for estimating bias [55,56], all available from the statistical software MedCalc^®^. As the hypothesis of normal distribution of measures was rejected, non-parametric tests (Wilcoxon test) were also used for comparing the results. If *p* < 0.05, then two means differed significantly.

## 5. Conclusions

The paper presents a validated simple, accurate and stable HPLC-UV method for the routine monitoring of MPA in human plasma. The methodology has been positively verified with several years of practical use for therapeutic drug monitoring, and therefore it can be recommended for medical (hospital) laboratories equipped with solely basic HPLC apparatus as an attractive alternative both for the LC-MS/MS technique and for immunochemical tests. At the same time, we showed that the procedure can be further simplified by disapplying an internal standard, which, paradoxically, may provide better specificity and precision of measurements.

## Figures and Tables

**Table 1:** Precision and accuracy of the method calculated with and without IS [intra-assay (*n* = 5); inter-assay (*n* = 4); LLOQ and QC samples: L, M, H (*n* = 3)].

MPA Concentration Added (µg/mL)	INTRA-ASSAY			INTER-ASSAY		
	Concentration Determined (Mean ± SD) (µg/mL)	Imprecision (RSD) (%)	Inaccuracy (%)	Concentration Determined (Mean ± SD) (µg/mL)	Imprecision (RSD) (%)	Inaccuracy (%)
0.1 (LLOQ)	0.115 ± 0.008 0.114 ± 0.010	6.63 8.42	+15.40 +14.30	0.119 ± 0.003 0.119 ± 0.004	2.62 3.32	+19.00 +18.72
0.3 (QC-L)	-	-	-	0.316 ± 0.010 0.307 ± 0.019	3.02 6.09	+5.31 +2.27
0.5	0.504 ± 0.036 0.482 ± 0.035	7.06 7.27	+0.86 −3.59	0.482 ± 0.020 0.479 ± 0.008	4.12 1.58	−3.63 −4.12
1	0.943 ± 0.026 0.980 ± 0.028	2.79 2.86	−5.72 −2.05	0.912 ± 0.047 0.922 ± 0.047	5.15 5.14	−8.82 −7.81
2	1.898 ± 0.034 1.971 ± 0.042	1.82 2.11	−5.09 −1.47	1.837 ± 0.077 1.856 ± 0.091	4.17 4.89	−8.15 −7.20
4 (QC-M)	-	-	-	3.832 ± 0.121 3.781 ± 0.121	3.17 3.20	−4.21 −5.47
5	4.875 ± 0.113 4.929 ± 0.044	2.31 0.90	−2.51 −1.42	4.848 ± 0.112 4.855 ± 0.101	2.31 2.07	−3.03 −2.90
10	9.735 ± 0.094 9.961 ± 0.150	0.97 1.51	−2.65 −0.39	9.882 ± 0.302 9.858 ± 0.076	3.06 0.77	−1.18 −1.42
20	19.708 ± 0.501 19.918 ± 0.449	2.54 2.26	−1.46 −0.41	20.054 ± 0.530 20.061 ± 0.096	2.64 0.48	+0.27 +0.30
25 (QC-H)	-	-	-	25.299 ± 0.658 24.702 ± 0.516	2.60 2.09	+1.20 −1.19
40 (ULOQ)	41.184 ± 0.852 40.884 ± 0.451	2.07 1.10	+2.96 +2.21	40.424 ± 0.774 40.448 ± 0.415	1.92 1.03	+1.06 +1.12

Numbers in upper line—results calculated with IS, numbers in lower line—results calculated without IS.

**Table 2:** Long-term stability (*n* = 3).

Storage Time at −24 °C (Weeks)	Low Concentration Calculated with and without IS (Mean ± SD) (µg/mL)	Stability for Low Concentration Calculated with and without IS (%)	High Concentration Calculated with and without IS (Mean ± SD) (µg/mL)	Stability for High Concentration Calculated with and without IS (%)
0 (initial)	1.135 ± 0.052 1.142 ± 0.034	100.00 100.00	24.492 ± 0.693 25.211 ± 0.692	100.00 100.00
1	1.166 ± 0.011 1.185 ± 0.019	102.80 103.77	24.487 ± 0.376 24.515 ± 0.447	99.98 97.24
2	1.132 ± 0.053 1.149 ± 0.025	99.74 100.61	23.874 ± 0.817 25.080 ± 0.580	97.47 99.48
3	1.294 ± 0.032 1.195 ± 0.017	114.07 104.69	25.797 ± 0.424 24.894 ± 0.909	105.33 98.74
4	1.169 ± 0.013 1.125 ± 0.018	103.03 98.57	25.248 ± 0.314 24.518 ± 0.341	103.08 97.25
5	1.297 ± 0.059 1.198 ± 0.061	114.31 104.95	25.506 ± 0.408 24.828 ± 0.421	104.14 98.48
6	1.276 ± 0.028 1.211 ± 0.039	112.46 106.07	26.161 ± 1.380 25.397 ± 0.790	106.81 100.74

Numbers in upper line—results calculated with IS, numbers in lower line—results calculated without IS.

**Table 3:** Freeze-thaw stability (*n* = 3).

Cycle/Storage Time (hours)	Low Concentration Calculated with and without IS (Mean ± SD) (µg/mL)	Stability for Low Concentration Calculated with and without IS (%)	High Concentration Calculated with and without IS (Mean ± SD) (µg/mL)	Stability for High Concentration Calculated with and without IS (%)
0 (initial)	1.334 ± 0.031 1.282 ± 0.037	100.00 100.00	26.504 ± 0.633 25.290 ± 0.802	100.00 100.00
72	1.320 ± 0.016 1.256 ± 0.038	98.93 98.00	26.518 ± 0.501 25.309 ± 0.576	100.05 100.07
144	1.301 ± 0.013 1.248 ± 0.043	97.51 97.30	26.028 ± 1.100 25.581 ± 1.028	98.21 101.15
216	1.221 ± 0.038 1.276 ± 0.061	91.48 99.55	24.399 ± 0.841 26.072 ± 0.904	92.06 103.09

Numbers in upper line—results calculated with IS, numbers in lower line—results calculated without IS.

**Table 4:** Short-term stability (*n* = 3).

Procedure Description	Low Concentration Calculated with and without IS (Mean ± SD) (µg/mL)	Stability for Low Concentration Calculated with and without IS (%)	High Concentration Calculated with and without IS (Mean ± SD) (µg/mL)	Stability for High Concentration Calculated with and without IS (%)
Standard analytical procedure	1.227 ± 0.070 1.196 ± 0.015	100.00 100.00	24.637 ± 0.796 25.209 ± 0.434	100.00 100.00
Supernatant stability	1.201 ± 0.049 1.199 ± 0.024	97.83 100.25	24.883 ± 0.567 24.991 ± 0.541	101.00 99.14
Dried extract stability	1.197 ± 0.029 1.212 ± 0.045	97.54 101.32	24.416 ± 0.808 24.881 ± 0.747	99.11 98.70
Reconstituted sample stability	1.239 ± 0.013 1.205 ± 0.047	100.93 100.76	25.634 ± 1.211 25.256 ± 0.594	104.05 100.19

Numbers in upper line—results calculated with IS, numbers in lower line—results calculated without IS.

**Table 5:** MPA concentrations in heart transplant patients samples (*n* = 1187).

Parameter	MPA Concentration Calculated with IS (µg/mL)	MPA Concentration Calculated without IS (µg/mL)
Range	0.16–9.49	0.16–9.54
Mean ± SD	1.99 ± 1.24	1.98 ± 1.22
Median	1.73	1.72

**Table 6:** MPA concentrations in spiked plasma samples (*n* = 45 + 45).

Parameter	Spiked Low MPA Concentration Calculated with IS (µg/mL)	Spiked Low MPA Concentration Calculated without IS (µg/mL)	Spiked High MPA Concentration Calculated with IS (µg/mL)	Spiked High MPA Concentration Calculated without IS (µg/mL)
Range	1.07–1.35	1.11–1.33	22.99–27.63	23.85–26.77
Mean ± SD	1.23 ± 0.07	1.21 ± 0.05	25.24 ± 1.05	25.14 ± 0.69
Median	1.23	1.21	25.10	25.19
RSD (%)	5.91	4.55	4.18	2.73

## Data Availability

Not applicable.
